# Supplementary figures and images for: scRNA-Seq reveals distinct stem cell populations that drive hair cell regeneration after loss of Fgf and Notch signaling
Source: eLife. 2019 Jan 25;8:e44431. doi: 10.7554/eLife.44431 (PMC6363392; doi:10.7554/eLife.44431)

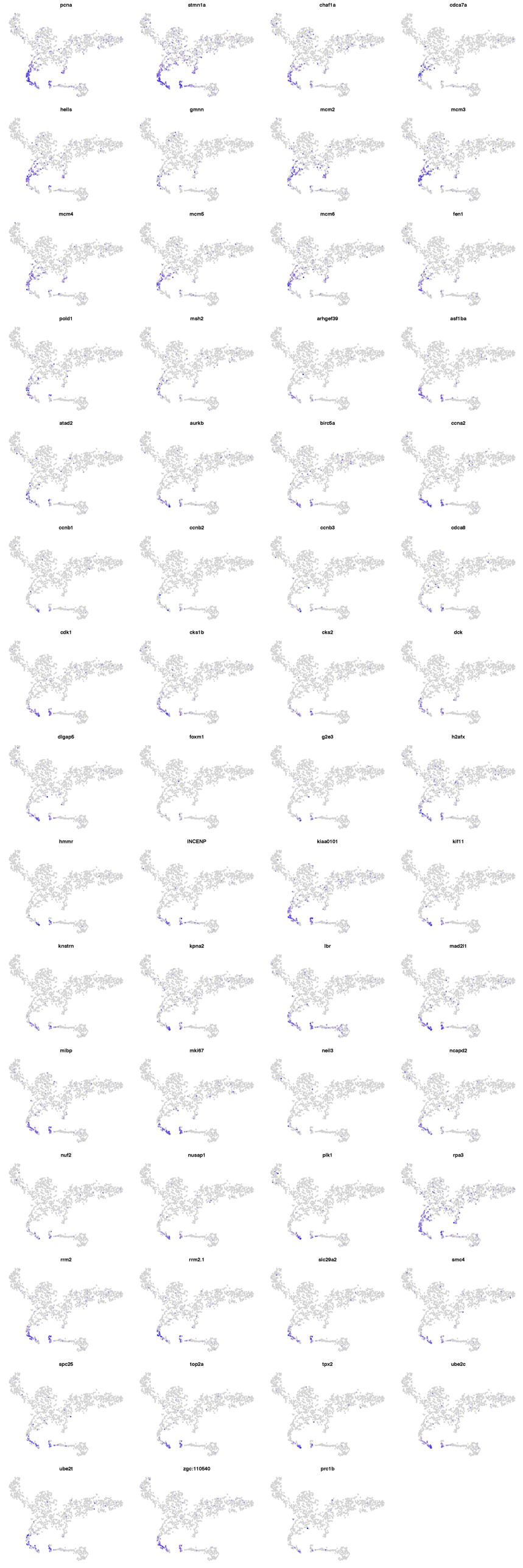

Supplement: Supplementary file 5. [file elife-44431-supp5.jpg]
